# Randomized feasibility trial of the Scleroderma Patient-centered Intervention Network Self-Management (SPIN-SELF) Program

**DOI:** 10.1186/s40814-022-00994-5

**Published:** 2022-02-26

**Authors:** Linda Kwakkenbos, Nora Østbø, Marie-Eve Carrier, Warren R. Nielson, Claire Fedoruk, Brooke Levis, Richard S. Henry, Janet Pope, Tracy Frech, Shadi Gholizadeh, Sindhu R. Johnson, Pamela Piotrowski, Lisa R. Jewett, Jessica Gordon, Lorinda Chung, Dan Bilsker, Lydia Tao, Kimberly A. Turner, Julie Cumin, Joep Welling, Catherine Fortuné, Catarina Leite, Karen Gottesman, Maureen Sauvé, Tatiana Sofia Rodriguez Reyna, Marie Hudson, Maggie Larche, Ward van Breda, Maria E. Suarez-Almazor, Susan J. Bartlett, Vanessa L. Malcarne, Maureen D. Mayes, Isabelle Boutron, Luc Mouthon, Andrea Benedetti, Brett D. Thombs, on behalf of the SPIN Investigators, Laura K. Hummers, Robert Riggs, Shervin Assassi, Ghassan El-Baalbaki, Carolyn Ells, Kim Fligelstone, Amy Gietzen, Geneviève Guillot, Daphna Harel, Monique Hinchcliff, Christelle Nguyen, François Rannou, Karen Nielsen, Michelle Richard, Anne A. Schouffoer, Christian Agard, Nassim Ait Abdallah, Alexandra Albert, Marc André, Elana J. Bernstein, Sabine Berthier, Lyne Bissonnette, Alessandra Bruns, Marion Casadevall, Benjamin Chaigne, Chase Correia, Benjamin Crichi, Robyn Domsic, James V. Dunne, Bertrand Dunogue, Regina Fare, Dominique Farge-Bancel, Paul R. Fortin, Brigitte Granel-Rey, Genevieve Gyger, Eric Hachulla, Ariane L. Herrick, Sabrina Hoa, Alena Ikic, Niall Jones, Nader Khalidi, Marc Lambert, David Launay, Hélène Maillard, Nancy Maltez, Joanne Manning, Isabelle Marie, Maria Martin, Thierry Martin, Ariel Masetto, François Maurier, Arsene Mekinian, Sheila Melchor, Mandana Nikpour, Louis Olagne, Vincent Poindron, Susanna Proudman, Alexis Régent, Sébastien Rivière, David Robinson, Esther Rodriguez, Sophie Roux, Perrine Smets, Vincent Sobanski, Robert Spiera, Virginia Steen, Evelyn Sutton, Carter Thorne, Pearce Wilcox, Angelica Bourgeault, Mara Cañedo Ayala, Andrea Carboni Jiménez, Marie-Nicole Discepola, Maria Gagarine, Julia Nordlund

**Affiliations:** 1 Department of Clinical Psychology, Radboud University, Montessorilaan 3, 6525 HR Nijmegen, The Netherlands; 2 Lady Davis Institute of the Jewish General Hospital, Montreal, Quebec Canada; 3 St. Joseph’s Health Care, London, Ontario Canada; 4 Centre for Prognosis Research, School of Medicine, Keele University, Staffordshire, UK; 5 Department of Psychiatry, McGill University, Montreal, Quebec Canada; 6 Department of Medicine, University of Western Ontario, London, Ontario Canada; 7 Department of Internal Medicine, University of Utah, Salt Lake City, UT USA; 8 California School of Professional Psychology/Alliant, Los Angeles, CA USA; 9 Toronto Scleroderma Program, Mount Sinai Hospital & Toronto Western Hospital, Toronto, Ontario Canada; 10 Institute of Health Policy, Management, and Evaluation, University of Toronto, Toronto, Ontario Canada; 11 Private practice – Nutrition, Hamilton, Ontario Canada; 12 Department of Psychology, Institute of Community and Family Psychiatry, Jewish General Hospital, Montreal, Quebec Canada; 13 Department of Medicine, Hospital for Special Surgery, New York City, NY USA; 14 Department of Medicine, Stanford University, Palo Alto, CA USA; 15 Department of Medicine Palo Alto VA Health Care System, Palo Alto, CA USA; 16 Faculty of Health Sciences, Simon Fraser University, Burnaby, British Columbia Canada; 17 Department of Psychiatry, University of British Columbia, Vancouver, British Columbia Canada; 18 NVLE Dutch patient organization for Systemic Autoimmune Diseases, Utrecht, The Netherlands; 19 Ottawa Scleroderma Support Group, Ottawa, Ontario Canada; 20 University of Minho, Braga, Portugal; 21 Scleroderma Foundation, Los Angeles, CA USA; 22 Scleroderma Canada, Hamilton, Ontario Canada; 23 Scleroderma Society of Ontario, Hamilton, Ontario Canada; 24 Instituto Nacional de Ciencias Médicas y Nutrición Salvador Zubirán, Mexico, Mexico; 25 Department of Medicine, McGill University, Montreal, Quebec Canada; 26 Department of Medicine, McMaster University and St Joseph’s Healthcare, Hamilton, Ontario Canada; 27 Faculty of Behavioural and Movement Sciences, Vrije University, Amsterdam, The Netherlands; 28 Department of General Internal Medicine, University of Texas MD Anderson Cancer Center, Houston, TX USA; 29 Centre for Outcomes Research and Evaluation, Research Institute of the McGill University Health Centre, Montreal, Quebec Canada; 30 Department of Psychology, San Diego State University, San Diego, CA USA; 31 Joint Doctoral Program in Clinical Psychology, San Diego State University/University of California San Diego, San Diego, CA USA; 32 Department of Internal Medicine, University of Texas McGovern School of Medicine, Houston, TX USA; 33 Université de Paris, Centre of Research Epidemiology and Statistics (CRESS), Inserm, INRA, Paris, France; 34 Centre d’Épidémiologie Clinique, Assistance Publique–Hôpitaux de Paris (AP-HP), Hôpital Hôtel Dieu, Paris, France; 35 Service de Médecine Interne, Centre de Référence Maladies Autoimmunes Systémiques Rares d’Ile de France, Hôpital Cochin, Assistance Publique-Hôpitaux de Paris (AP-HP), Paris, France; 36 APHP-CUP, Hôpital Cochin, Université de Paris, F-75014 Paris, France; 37 Department of Epidemiology, Biostatistics, and Occupational Health, McGill University, Montreal, Quebec Canada; 38 Respiratory Epidemiology and Clinical Research Unit, McGill University Health Centre, Montreal, Quebec Canada; 39 Department of Psychology, McGill University, Montreal, Quebec Canada; 40 Department of Educational and Counselling Psychology, McGill University, Montreal, Quebec Canada; 41 Biomedical Ethics Unit, McGill University, Montreal, Quebec Canada

**Keywords:** Feasibility trial, Scleroderma, Systemic sclerosis, Self-management, Internet intervention, Cohort multiple RCT

## Abstract

**Background:**

The Scleroderma Patient-centered Intervention Network (SPIN) developed an online self-management program (SPIN-SELF) designed to improve disease-management self-efficacy in people with systemic sclerosis (SSc, or scleroderma). The aim of this study was to evaluate feasibility aspects for conducting a full-scale randomized controlled trial (RCT) of the SPIN-SELF Program.

**Methods:**

This feasibility trial was embedded in the SPIN Cohort and utilized the cohort multiple RCT design. In this design, at the time of cohort enrollment, cohort participants consent to be assessed for trial eligibility and randomized prior to being informed about the trial. Participants in the intervention arm are informed and provide consent, but not the control group. Forty English-speaking SPIN Cohort participants from Canada, the USA, or the UK with low disease-management self-efficacy (Self-Efficacy for Managing Chronic Disease Scale [SEMCD] score ≤ 7) who were interested in using an online self-management program were randomized (3:2 ratio) to be offered the SPIN-SELF Program or usual care for 3 months. Program usage was examined via automated usage logs. User satisfaction was assessed with semi-structured interviews. Trial personnel time requirements and implementation challenges were logged.

**Results:**

Of 40 SPIN Cohort participants randomized, 26 were allocated to SPIN-SELF and 14 to usual care. Automated eligibility and randomization procedures via the SPIN Cohort platform functioned properly, except that two participants with SEMCD scores > 7 (scores of 7.2 and 7.3, respectively) were included, which was caused by a system programming error that rounded SEMCD scores. Of 26 SPIN Cohort participants offered the SPIN-SELF Program, only 9 (35%) consented to use the program. Usage logs showed that use of the SPIN-SELF Program was low: 2 of 9 users (22%) logged into the program only once (median = 3), and 4 of 9 (44%) accessed none or only 1 of the 9 program’s modules (median = 2).

**Conclusions:**

The results of this study will lead to substantial changes for the planned full-scale RCT of the SPIN-SELF Program that we will incorporate into a planned additional feasibility trial with progression to a full-scale trial. These changes include transitioning to a conventional RCT design with pre-randomization consent and supplementing the online self-help with peer-facilitated videoconference-based groups to enhance engagement.

**Trial registration:**

clinicaltrials.gov, NCT03914781. Registered 16 April 2019.

## Key messages regarding feasibility

- What uncertainties existed regarding the feasibility?

An online, self-guided, self-management intervention to support self-efficacy for disease management in people with systemic sclerosis (SSc) was developed (SPIN-SELF Program). The planned full-scale randomized controlled trial (RCT) would be conducted using the novel cohort multiple RCT (cmRCT) design. In the cmRCT design, compared with conventional parallel-group RCT designs, randomization to the intervention or control arm is conducted prior to obtaining consent for the intervention, thus declining participation happens post-randomization. Uptake of the intervention offer has been low in previous studies with the cmRCT design. Thus, prior to testing for effectiveness in a full-scale RCT, we sought to (1) evaluate the offer uptake of the intervention as well as other aspects of feasibility of the planned trial methodology, and (2) assess whether the online intervention is user-friendly and acceptable to trial participants.

- What are the key feasibility findings?

Forty participants were enrolled in the SPIN-SELF feasibility trial, and the randomization feature embedded in the Cohort platform functioned properly. Participants were able to easily connect to and use the online SPIN-SELF Program and found the program easy to use. Two key problems related to the feasibility of conducting a full-scale RCT of the SPIN-SELF Program were identified: (1) of 26 participants offered to try the SPIN-SELF Program, only 9 (35%) consented to use it, and (2) usage logs showed that use of the SPIN-SELF Program was low: 2 of 9 users (22%) logged into the program only once (median = 3), and 4 participants (44%) accessed none or only 1 of the 9 available modules in the program (median = 2).

- What are the implications of the feasibility findings for the design of the main study?

The design of the main study needs to be adapted to improve enrollment in the SPIN-SELF Program as well as usage of the program among trial participants. Changes will include a transition from the cmRCT design to a conventional parallel-group RCT design and peer-facilitated online groups to support engagement with the SPIN-SELF Program will be organized in addition to the online self-guided program.

## Background

Self-management programs are designed to support people with chronic medical conditions to improve their self-efficacy to manage their condition, maintain life roles, and cope with negative emotions. They are typically delivered in group formats and emphasize the development of problem-solving skills and effective collaboration with health care providers [1–3]. General and disease-specific self-management programs have been shown to improve self-efficacy for disease management, and health and quality of life outcomes (e.g., [4–10]), although self-guided online programs may not be effective [11]. Generic programs and programs developed for common conditions, however, do not address the unique challenges of people with rare diseases [12].

Rare diseases are defined as conditions that affect fewer than 1 in 2000 people. Cumulatively, 4–6% of people may have a rare disease [13]. Systemic sclerosis (SSc, or scleroderma) is a rare, chronic, autoimmune disease characterized by vasculopathy and excessive collagen production; it affects the skin and internal organs, including the lungs, gastrointestinal tract, and cardiovascular system [14]. Common problems include physical mobility and hand function limitations, gastrointestinal problems, fatigue, pain, sexual dysfunction, and body image distress from disfiguring changes in appearance [15, 16]. One randomized controlled trial (RCT; *N* = 267) tested a SSc internet-based self-management program but reported that it did not improve self-efficacy or other outcomes [17].

The Scleroderma Patient-centered Intervention Network (SPIN) is an international collaboration of researchers, clinicians, patient advocates, and people with SSc that develops, tests, and disseminates tools to support people living with SSc [18]. Patient collaborators prioritized the development of a self-management program and jointly designed the SPIN-SELF Program with SPIN investigators. It was designed as a self-guided, online intervention in order to facilitate access and dissemination of knowledge and resources in a rare disease environment, since many people with SSc do not live near specialized SSc treatment centers.

The aim of the SPIN-SELF feasibility trial was to inform necessary adjustments to the program and trial procedures prior to conducting a full-scale RCT by assessing trial procedures, required resources and management, scientific aspects, and participant acceptability of procedures and program content.

## Methods

### Design and setting

The SPIN-SELF Feasibility Trial was a parallel, two-arm feasibility cohort multiple RCT (cmRCT) conducted using the SPIN Cohort to identify and enroll eligible participants and ascertain outcomes. In the cmRCT design [19], participants are randomized to be offered an intervention or not based on eligibility criteria applied to their regular cohort assessments, prior to being notified about the trial. Those randomized to be offered the intervention must consent to receive it. Those not offered the intervention are not notified about the trial and only complete regular cohort measures. The trial was registered (NCT03914781), and a protocol was published [20]. Results are reported in accordance with the Consolidated Standards of Reporting Trials (CONSORT) extension for randomized pilot and feasibility trials [21] and the CONSORT extension for trials using cohorts and routinely collected data [22]. There were no changes to the protocol after the trial commenced. Ethics approval for the SPIN Cohort was obtained from the Research Ethics Committee of the Centre intégré universitaire de santé et de services sociaux du Centre-Ouest-de-l'Île-de-Montréal (#MP-05-2013-150) and all participating SPIN centers. Approval for the SPIN-SELF Feasibility Trial was obtained from the Research Ethics Committee of the Centre intégré universitaire de santé et de services sociaux du Centre-Ouest-de-l’Île-de-Montréal (#2019-1146).

### SPIN Cohort participants

The SPIN Cohort [18] has collected patient-reported outcomes at 3-month intervals via the internet since April 2014. Eligible participants must be classified as having SSc based on the 2013 American College of Rheumatology/ European League Against Rheumatism (ACR/EULAR) criteria [23], confirmed by a SPIN physician; ≥ 18 years old; and fluent in English, French, or Spanish. SPIN Cohort participants are recruited at 50 SPIN sites (https://www.spinsclero.com/en/cohort/sites) during regular medical visits, and written informed consent is obtained. Participants consent to (1) their data being used for observational studies; (2) their data being used to assess intervention trial eligibility and, if eligible, that they will be randomized; (3) if eligible and randomized to the intervention arm of a trial, to be contacted and offered access to the intervention; and (4) if eligible and randomized to the usual care arm of a trial, to not be notified about the trial but to use their regularly collected cohort data to evaluate trial outcomes. A medical data form is completed by the treating physician and submitted online to enroll participants. Approximately 1300-1500 active participants from 7 countries and 50 sites complete assessments in any 3-month period.

### SPIN-SELF feasibility trial eligibility

Trial eligibility assessments occurred automatically via the online SPIN Cohort platform during participants’ regular SPIN Cohort assessments. Eligible participants were those who completed cohort measures in English, had low disease management self-efficacy (score ≤ 7 on the Self-Efficacy for Managing Chronic Disease Scale (SEMCD) [24]), and indicated high interest in using an online self-management intervention (≥ 6 on 0-10 scale).

### Procedure: randomization, allocation concealment, consent, and blinding

We used simple randomization of participants with a 3:2 ratio to an offer to use the SPIN-SELF Program for 3 months versus usual care. A 3:2 ratio was used to attempt to enroll enough participants who would be offered the intervention to assess usage. Eligible participants were identified and randomized automatically as they completed regular SPIN Cohort assessments using a feature in the SPIN Cohort platform, which provides immediate centralized randomization and, thus, complete allocation sequence concealment.

Participants randomized to be offered the program received an automated email invitation including a link to the SPIN-SELF Program website and the consent form. At initial login, using their SPIN Cohort username and password, they were prompted to provide written consent to participate in testing the SPIN-SELF Program by verifying agreement with consent elements and providing their email address as electronic signature. Consented participants were automatically directed to the SPIN-SELF Program introduction page. Participants who logged out without consenting were returned to the consent page upon subsequent logins. Participants who accepted the offer could use the web link to enter the secure intervention site for 3 months.

SPIN personnel also contacted participants by phone, usually within 48 hours of sending the invitation email, to describe the study, review the consent form, and answer questions. A maximum of 5 attempts were made to contact participants within 10 days of sending the invitation email. If not successfully contacted, a sixth attempt was made approximately 20 days post-invite. Email and phone technical support were available to help participants with the consent process and use of the intervention site.

Participants assigned to usual care were not notified about the trial and completed their regular SPIN Cohort assessments as normal. Thus, participants offered the intervention were not blind to their status, whereas participants assigned to usual care were blind to their trial participation and assignment. This replicates actual practice, where patients are not typically advised about treatments that are not options and may reduce risk of disappointment bias [19, 25].

### Intervention and comparator

The SPIN-SELF Program was designed based on key tenets of self-management skills that have been incorporated in successful self-management programs for chronic diseases, including problem solving, decision making, resource utilization, forming a patient–health care provider partnership, and taking action [26]. Input on modules, content, layout, and program navigation was obtained from focus groups with people with SSc and SPIN’s Patient Advisory Board [27].

Upon first use, instructions on how to navigate the program are provided in a website tour video. Participants are introduced to the concept of self-management by a physician with expertise in SSc and a patient sharing her experience with self-management. The program utilizes an engaging and easy-to-navigate web interface, and language of the modules is at an appropriate level for understanding based on input from members of SPIN’s Patient Advisory Board. Pages can be bookmarked, text can be enlarged, and captions for each video are available for download. There are 9 modules, and after the program introduction, users are directed to a 9-item quiz designed to guide them to modules most relevant to their symptoms and disease-management challenges. Based on the quiz score, links to the 3 most relevant modules appear on top, with links to the other 6 modules available below. The 9 modules target (1) coping with pain; (2) skin care, finger ulcers, and Raynaud’s; (3) sleep problems; (4) fatigue; (5) gastrointestinal symptoms; (6) itch; (7) managing emotions and stress; (8) coping with body image concerns due to disfigurement; and (9) effective communication with healthcare providers. Each module includes an educational component that provides background information on the topic and teaching of skills, as well as a goal-setting component.

In addition to the modules, the SPIN-SELF Program includes tools to support key self-management strategies, including education on goal setting, fillable goal-setting forms, and worksheets to help users integrate newly learned skills and techniques into daily routines. The online goal form encourages users to choose a number of days per week and total weeks to work on a goal, to reflect on why achieving the goal is important to them, and to write their reason in a text box. For each goal, users can input weekly progress, receive e-mail reminders to facilitate successful completion, and share goals and progress with friends and family. Worksheets are specifically tailored to each module and can be saved and printed for later use. The program incorporates social modeling through educational videos of people with SSc who describe their challenges and coping strategies, and of health professionals who teach key self-management techniques.

### Feasibility outcomes

Feasibility outcomes included: [1] proportion of SPIN Cohort participants who met eligibility criteria [2]; functioning of automated eligibility assessment and randomization procedures [3]; proportion of eligible participants randomized to the intervention arm who consented to participate [4]; completeness of online data collection for each trial arm at 3-month follow-up [5]; completeness of intervention usage log data [6]; ability to successfully link data from the SPIN Cohort and SPIN-SELF platforms using person-level deterministic linkage with participant SPIN identification numbers [7]; personnel requirements to support consent and use of the program [8]; technological performance of the online SPIN-SELF Program; and [9] any other unanticipated challenges. Usage data provided detailed information on the number of logins, the number of modules accessed, and goals set. The data were linked to SPIN Cohort data using participants’ SPIN-ID numbers, which were used for both platforms. Study personnel tracked their activities and the time spent on an Excel spreadsheet.

To assess user acceptability and satisfaction, semi-structured interviews were conducted with consenting participants in the intervention arm, at 3 months post-randomization, using 27 items of the Patient Education Materials Assessment Tool for Audiovisual Materials (PEMAT) [28], which addresses program usability, understandability, organization, and clarity (see Appendix for the interview guide).

### SPIN-SELF planned trial outcome measures

Measures of disease-management self-efficacy and functional health outcomes that will be used to evaluate the program in the full-scale trial were reported, although no hypothesis tests were conducted, consistent with the feasibility trial design [21, 29]. Outcomes were collected as part of each participant’s routine SPIN Cohort assessments 3 months post-randomization. The planned primary outcome for the SPIN-SELF full-scale trial is the SEMCD.

The 6-item SEMCD [24] measures respondent confidence to manage fatigue, pain, emotional distress, and other symptoms; to do things other than take medication to reduce illness impact; and to carry out tasks that may reduce the need to see a doctor. Items are rated on a 1 (*not confident at all*) to 10 (*totally confident*) scale. The total score is the mean of all items. The SEMCD was previously validated for SSc through the SPIN Cohort [30].

The 29-item Patient-Reported Outcomes Measurement Information System (PROMIS-29) profile version 2.0 [31] assesses 8 domains of health status (depression symptoms; anxiety symptoms; physical function; pain interference; fatigue; sleep disturbance; ability to participate in social roles and activities; pain intensity); there are 4 items in each of 7 domains plus a single item for pain intensity. Raw scores are converted into *T*-scores standardized from the general US population (mean = 50, standard deviation = 10). Higher scores indicate greater physical function and social role and activity participation but worse anxiety, depression, fatigue, sleep disturbance, pain interference, and pain intensity. The PROMIS-29 version 2.0 has been validated for SSc [32, 33].

### Sample size

Guidance on the appropriate sample size for feasibility trials varies, with rules-of-thumb suggesting 12 to 30 participants or more per trial arm [34, 35]. In a previous SPIN feasibility trial of a hand exercises intervention, offer acceptance rate was approximately 60% [36]. We included a total of 40 SPIN Cohort participants with 3:2 randomization to support additional information needs from intervention arm participants.

### Statistical analysis

Descriptive statistics were used to characterize the sample and feasibility outcomes, including personnel and time resources required, participant eligibility, recruitment numbers, percentages of participants who responded to follow-up measures, and frequency of logins and time spent using the program. The presence of floor or ceiling effects on outcome measures was assessed. Qualitative information on participant experiences was recorded.

### Criteria for progression to full-scale trial

Feasibility outcomes were evaluated qualitatively, and no quantitative cut-offs for progression to full-scale trial (e.g., % consent) were pre-specified.

## Results

### Participant characteristics

**Fig. 1:**
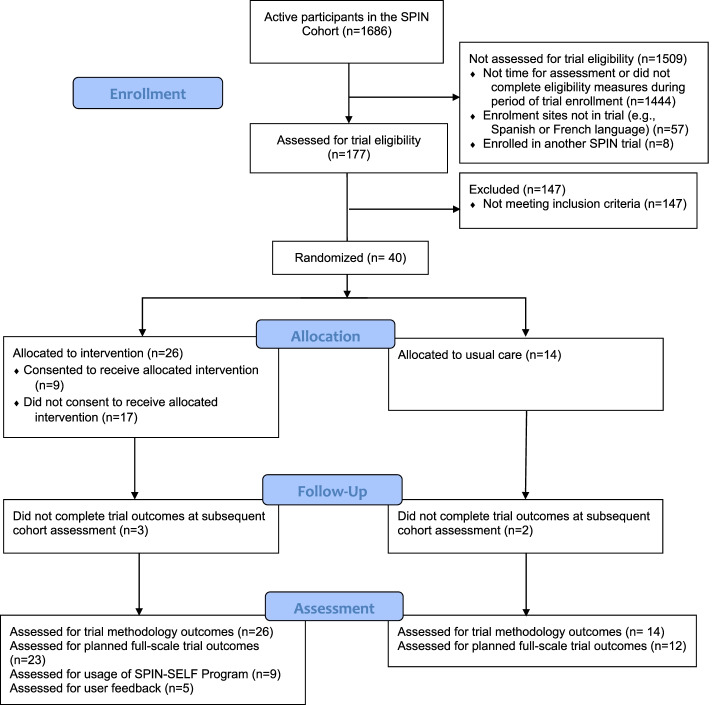
Flow diagram for the SPIN-SELF feasibility trial

Enrollment began on July 6, 2019 and was completed on July 27, 2019. Of 40 participants, 26 (65%) were allocated to the intervention arm and 14 (35%) to the usual care arm (see Fig. 1 for SPIN Cohort participant flow through the SPIN-SELF feasibility trial). Demographic, disease characteristics, SEMCD Scale, and PROMIS-29 scores for both groups are shown in Table 1.

**Table 1:** Demographic and disease characteristics (*N* = 40)

Variable	SPIN-SELF	Usual care
	***N*** = 26	***N*** = 14
**Demographic**		
Age in years, mean (SD)	54.7 (11.3)	62.9 (7.3)
Female sex, *N* (%)	24 (92.3)	13 (92.9)
Education in years, mean (SD)	15.6 (3.1)	15.6 (3.0)
Married or living as married, *N* (%)	19 (73.1)	10 (71.4)
Race/ethnicity, *N* (%)		
White	21 (80.8)	12 (85.7)
Black	2 (7.7)	2 (14.3)
Other	3 (11.5)	0 (0.0)
Country, *N* (%)		
Canada	9 (34.6)	4 (28.6)
The USA	12 (46.2)	7 (50.0)
The UK	5 (19.2)	3 (21.4)
**Disease characteristics**^**a**^		
Time since onset first non-Raynaud’s symptom or sign in years, mean (SD)	16.7 (9.3)^b^	12.7 (7.3)^c^
Time since diagnosis in years, mean (SD)	13.0 (8.3)^d^	10.6 (5.2)
Diffuse disease subtype, N (%)	11 (44.0)^e^	8 (50.0)
**Self-Efficacy for Managing Chronic Disease Scale** score (SEMCD)		
SEMCD Scale score, mean (SD)	5.0 (1.5)	4.8 (1.2)
**PROMIS-29**		
Physical function score, mean (SD)	39.2 (8.1)^e^	39.6 (9.9)
Anxiety score, mean (SD)	56.8 (8.7)^e^	53.1 (9.1)
Depression score, mean (SD)	54.9 (9.5)^e^	53.2 (9.0)
Fatigue score, mean (SD)	63.2 (8.1)^e^	57.8 (11.8)
Sleep disturbance score, mean (SD)	55.4 (7.2)^e^	52.4 (6.7)
Social roles and activities score, mean (SD)	40.7 (5.9)^e^	43.8 (11.5)
Pain interference score, mean (SD)	58.5 (7.0)^e^	61.8 (7.8)
Single item for pain intensity, mean (SD)	4.8 (2.2)^e^	4.9 (2.6)

^a^Disease characteristics were recorded at time of enrolment in the SPIN Cohort

Due to missing data: ^b^*N* = 20, ^c^*N* = 11, ^d^*N* = 24, ^e^*N* = 25

*N*, number; *PROMIS*, 29-item Patient-Reported Outcomes Measurement Information System; *SEMCD*, Self-Efficacy for Managing Chronic Disease Scale; *SD*, standard deviation

### Feasibility outcomes

During the enrollment period, 177 SPIN Cohort participants completed cohort assessments and were evaluated for eligibility. Of these, 40 (23%) met trial eligibility criteria. One error in the automated eligibility assessment process was identified. Two enrolled participants had SEMCD scores > 7 (7.2 and 7.3) and, thus, were randomized despite not meeting eligibility criteria. This occurred because of a system programming error that rounded scores rather than including decimal places. A review of participant characteristics and assessment scores showed that all other eligible and ineligible cohort participants were correctly classified.

Total time spent by SPIN personnel on calls, including leaving voicemails, and emails with the 26 participants randomized to the intervention arm was 228 min (8.8 min per participant). Participants who attempted to log in were able to do so easily, so no troubleshooting outside of the protocol calls was needed. One participant reported difficulties using the program on their smartphone but was able to successfully use it on a computer without assistance.

Study personnel spent an additional 99 min on other tasks, including reviewing the randomization process, tracking enrollment and consent progress, and managing call logs. Mean time per day was less than 5 min. This did not include team meetings to review project progress and administration. One technical issue was identified on the first day of enrollment when intervention participants did not appear on the SPIN Cohort platform’s management page. Time spent to resolve this issue by information technology personnel was 35 min. No other challenges were reported by study personnel.

### Consent and usage of the SPIN-SELF Program

Usage data from the SPIN-SELF platform and SPIN Cohort data were successfully linked 100% of the time. Of the 26 participants randomized to intervention, 15 (58%) did not log in to the consent page; 2 (8%) logged in but did not consent, and 9 (35%) consented. Of the 9 who consented, 2 (22%) logged in only once, 2 (22%) logged in twice, and the other 5 logged in 3 to 7 times (mean = 3.3; standard deviation = 2.0; median = 3).

After consent, 6 of 9 participants (67%) watched the expert introduction video, and 3 of these also watched the patient introduction (33%). Four participants (44%) used the website tour. All 9 participants completed the quiz to identify the modules most relevant to them. One participant (11%) did not access any module in the SPIN-SELF Program, and 3 participants (33%) accessed 1 of the 9 modules. Other participants accessed between 3 and 9 modules (overall mean = 2.7, standard deviation = 2.5, median = 2). Of the different modules, most participants (*N* = 6, 67%) accessed the fatigue module, 4 (44%) accessed the gastrointestinal symptoms module, and 3 participants (33%) accessed modules on sleep and pain. The modules addressing body image concerns, itch, and skin care were accessed by 2 participants each (22%), and the modules on effective communication with healthcare providers and managing emotions were accessed by 1 of the 9 participants (11%). Two participants (22%) created one or two goals, others did not use this feature.

### Interview outcomes/user feedback

Of the 9 intervention arm participants who consented to use the program, 5 participated in post-trial interviews. Of the remaining 4 participants who were not interviewed, 2 did not respond to attempts to contact, and 2 declined to be interviewed (one reported they did not use the program enough, one indicated being unable to due to health issues). See PEMAT interview responses in Table 2. Overall, feedback of the 5 interviewed participants was very positive. The overall mean grade given by participants for the SPIN-SELF Program was 8.4/10. No concerns related to adverse events were reported.

**Table 2:** Summary of responses to the Patient Education Materials Assessment Tool for Audiovisual Materials (PEMAT) interviews

PEMAT item	Summary of responses
**General**	
Did you use a computer or tablet or both to access the SPIN-SELF Program? Can you please tell us about your experience with the SPIN-SELF Program, including things that you liked about the program and things that could be improved?	4 computers, 1 tablet (with occasional computer use). 1 liked the program very much; 1 liked the various areas covered by the program, it gives the user choices on what they want to focus on based on their experience with the disease; 1 learned a lot from the fatigue module (pacing activities); 1 liked the expert and patient videos because they offer different perspectives, could improve length of modules (some are wordy); 1 found the format really easy to use and the information extremely helpful, enjoyed the worksheets but was unable to use them on a tablet.
**Process**	
Did the initial invitation email provide you with the information you needed to understand how to sign up for the study? *If no, what information was missing?*	5 yes.
Did you find the follow up telephone call you received within 48 hours of the invitation email to be helpful? *If no, why not?*	5 yes.
**Purpose**	
Did you understand the objective of the SPIN-SELF program? *If no, how could the objective be clarified?*	5 yes.
Did you find the information provided in the SPIN-SELF program relevant? *If no, how could the information provided be made more relevant for you or other scleroderma patients?*	4 yes; 1 found the information provided by the module on digestive system and nutrition irrelevant to her gastro-intestinal issues.
**Words and language**	
Did you find that the intervention used common, everyday language that was easy to understand? *If no, can you give an example of something or some word(s) that you did not understand?*	4 yes; 1 yes, but maybe not for someone who is newly diagnosed.
Did you understand all the medical terms or, if not, were they clearly explained in the SPIN-SELF program? *If no, can you give an example of medical term(s) that you did not understand?*	4 yes; 1 yes, but might be overwhelming for newly diagnosed.
**Content, organization, navigation**	
Did you find that the SPIN-SELF program is broken down into manageable chunks or sections? *If no, which parts of the content weren’t broken down into manageable chunks or sections and how could we improve them?*	5 yes.
Did you find the different pages or sections of the program to be clearly indicated? *If no, what section(s) could be more clearly labelled?*	5 yes.
Did you find it easy to navigate through the intervention and to understand where to go next? *If no, how could the different steps to navigate the intervention be more clearly explained?*	4 yes; 1 yes, apart from a few times when using a tablet.
Did you consult the “More info” tab (Scleroderma 101, Patient Stories)? *If no, why not?*	4 yes; 1 no, has had the disease for 30 years so did not find it necessary to consult.
Did you experience any technical difficulties while using the intervention? *If yes, what type of technical problems? Did you request assistance from the SPIN team? If you did, was the SPIN team able to help you resolve them?*	4 no; 1 technical problems using tablet, spoke with SPIN team during a regular protocol call and they resolved the issue.
Did you use the website tour? *If yes, was it helpful to learn to navigate the website? Why or why not?*	1 yes, very helpful; 2 yes, but did not really need it/pay attention to it; 2 no.
Did you use the “My bookmarks” feature? *If yes, did you find it helpful for easily navigating to the pages you wanted? Why or why not?*	4 no; 1 yes, tried on tablet and found it difficult to use.
**Learning aids**	
Did the fact that the intervention was introduced by scleroderma experts and patients make the program more relatable? *Why or why not?*	2 yes, interesting to hear both perspectives; 1 yes, makes it more credible; 1 yes, for some modules but less for others (digestive system and nutrition); 1 yes.
Did you understand how to correctly use the techniques explained in the modules? *If no, what would have helped you better understand how to correctly use the techniques?*	5 yes.
Were you able to clearly understand the people speaking in the videos*? If no, why couldn’t you understand the words in the videos? (e.g. too fast, too soft, mumbling, accent)? Are there any videos in particular that were more difficult to understand than others? If yes, which one(s)?* Did you look at the video transcripts? *If yes, were they helpful? If yes, were the video transcripts helpful to you? Why or why not?*	2 yes; 2 yes, videos are clear and easy to understand; 1 didn’t really watch videos 3 no; 1 yes, very helpful; 1 yes, good to have them for more visual people.
**Actionability (worksheets, goal-setting, motivation)**	
Did you use the worksheets? *If no, why not? What could have been a better tool?* Did you set goals for yourself using the goal setting material? *Why or why not?*	3 yes (used them frequently—still uses them; used a couple; used one or two); 2 no. 3 yes (useful reminder; wanted to try it; helpful to achieve goals – although some goals were difficult to follow); 2 no (already has a good routine; does not remember that it was an option).
Did you use the option to share your goals with friends and family via email? *If yes, did the option to share your goals with friends and family via email help you stick to your goals? If no, what other motivational feature might have been more helpful?* Did you incorporate the tools and techniques you learned into your planned routine and stick to it? *If No: What were some obstacles you faced when trying to incorporate the tools and techniques into your routine? How could the SPIN-SELF program have helped you to overcome these obstacles?* Did you use the feature to track your progress? *If yes, did having the option to track your progress week after week encourage you to continue performing the techniques? If no, why not? Did you use any other way to track your progress? If so, what did you do?*	1 yes, important feature to link medical world and family – suggestion to add testimonies of past SPIN-SELF participants who followed the techniques and experienced improvements; 1 no, the program itself is very motivational and positive; 1 no, the usefulness of motivational features depends on the individual; 2 no - no other suggestion. 1 yes, some techniques have really been helpful; 1 yes, although sometimes it was difficult to stick to the routine; 1 no, was already equipped with techniques; 1 no, tried to incorporate some but did not stick to it – main obstacle was the number of medical appointments; 1 no – main obstacle was her habit of not giving herself time to rest and the program being online didn’t make a difference for her. 3 no, other personal ways to keep progress; 1 no, difficulty to use this feature on a tablet; 1 no.
Did you set email reminders for yourself? *If yes, did having the option set email reminders for yourself help you incorporate the techniques into your routine? If no, did you use another type of reminder?*	1 yes, very helpful; 2 no, did no use any type of reminder; 1 no, used calendar and alarm; 1 no, used a journal.
**Overall appreciation**	
How user-friendly on a 0–10 scale (0, being the worst and 10 being the best possible score) would you rate the SPIN-SELF program? Would you recommend this program to someone with scleroderma? *If no, why?* What grade (on a 0-10 scale, 0 being the worst and 10 being the best possible score) would you give the program? Is there anything you want to give us feedback about that was not included in this interview?	2 rated 10; 2 rated 8; 1 rated 5 for use on a tablet, but would have rated much higher if had been able to use the program on a computer. 5 yes. 2 rated 10; 1 rated 9; 1 rated 7; 1 rated 6. 2 no; 1 the health care module made a huge different for her and her confidence in tackling the healthcare system; 1 there is a lot of information and would have liked more guidance on what to focus on; 1 sections on finger ulcers and proton pump inhibitors could be included.

### SPIN-SELF planned trial outcome measures

Of the 40 participants, 35 (87.5%) completed their 3-month follow-up cohort assessments, including 23 in the intervention arm (88.5%) and 12 in the control arm (85.7%). Table 3 shows the SEMCD (planned primary outcome) and PROMIS-29 scores for both groups at baseline and 3-months follow-up, which are the planned trial outcome measures for the full-scale trial. The SPIN-SELF Feasibility Trial was not designed for hypothesis testing or effect size estimation, and the sample size was not appropriate to do so.

**Table 3:** Pre- and post-intervention total scores for the SEMCD and PROMIS-29v2 domains

Measure	Intervention ***N*** with data	Intervention mean (SD)	Control ***N*** with data	Control mean (SD)
SEMCD				
Baseline	26	5.0 (1.5)	14	4.8 (1.2)
Month 3	23	5.8 (1.5)	12	5.6 (1.5)
PROMIS-29 v2.0 physical function				
Baseline	25	39.2 (8.10)	14	39.6 (9.9)
Month 3	23	40.1 (8.9)	12	40.6 (9.4)
PROMIS-29 v2.0 anxiety				
Baseline	25	56.8 (8.7)	14	53.1 (9.1)
Month 3	23	54.5 (10.3)	12	51.0 (8.5)
PROMIS-29 v2.0 depression				
Baseline	25	54.9 (9.5)	14	53.2 (9.0)
Month 3	23	52.8 (10.3)	12	52.5 (8.0)
PROMIS-29 v2.0 fatigue				
Baseline	25	63.2 (8.1)	14	57.8 (11.8)
Month 3	23	61.4 (9.7)	12	54.6 (9.1)
PROMIS-29 v2.0 sleep disturbance				
Baseline	25	55.4 (7.2)	14	52.4 (6.7)
Month 3	23	55.5 (7.9)	12	53.6 (5.6)
PROMIS-29 v2.0 social roles				
Baseline	25	40.7 (5.9)	14	43.8 (11.5)
Month 3	23	40.9 (8.1)	12	46.5 (7.6)
PROMIS-29 v2.0 pain interference				
Baseline	25	58.5 (7.0)	14	61.8 (7.8)
Month 3	23	58.0 (8.2)	12	58.0 (7.7)
PROMIS-29 v2.0 pain intensity				
Baseline	25	4.8 (2.2)	14	4.9 (2.6)
Month 3	23	4.1 (2.4)	12	5.2 (2.3)

*N* number, *PROMIS* 29-item Patient-Reported Outcomes Measurement Information System, *SEMCD* Self-Efficacy for Managing Chronic Disease Scale, *SD* standard deviation

## Discussion

Forty SPIN Cohort participants were successfully enrolled in the SPIN-SELF feasibility trial, and the randomization feature embedded in the Cohort platform functioned properly. However, the automated eligibility procedure allowed for two ineligible participants to be enrolled. This was caused by a system programming error that rounded down the participants’ mean scores on the SEMCD Scale. This system error will be fixed to ensure that only eligible participants are randomized for the full-scale trial. Overall, the required trial personnel resources for the trial were low and mainly spent on follow-up calls made to enrolled participants. Participants were able to easily connect to and use the program, thus required technical support was minimal. Although only 5 trial participants in the SPIN-SELF arm completed the semi-structured interview, overall, they reported high satisfaction with the trial procedures as well as the SPIN-SELF Program (average score 8.4/10). They found the program easy to use and said they would recommend it to other people with SSc. One participant suggested that there is a lot of information and would have liked more guidance on what to focus on. The satisfaction estimate should be interpreted with caution, as it might be biased and possibly overestimates satisfaction, as patients who participated in the interview might rate the program more favorably than the ones not participating. The majority of the feasibility trial participants in both arms completed the 3-months follow-up assessment (87.5%). Two major problems related to the feasibility of conducting a full-scale RCT of the SPIN-SELF Program were identified, however. These include (1) the low consent rate among participants who were offered the SPIN-SELF Program (35%) and (2) the low usage of the program among consented participants.

In the cmRCT design, compared with conventional parallel-group RCT designs, randomization to the intervention or control arm is conducted prior to obtaining consent for the intervention, thus decisions to accept or decline participation in the intervention arm happen post-randomization [22]. Although there are currently few published complete trials using the cmRCT design, the acceptance rate of the intervention offered in published trials has consistently been low (40-50%) [22, 37, 38]. This was also the case in a recently completed trial of SPIN’s online hand exercises program (*N* = 466; consent rate 61%), which similarly enrolled participants through the SPIN Cohort [39]. Although the uptake of the SPIN-HAND Program was somewhat higher than previously published studies and the present feasibility trial (35%), the percentage of participants with uptake of the intervention offer in other trials using the cmRCT design appears to be problematic generally. When the cmRCT design was introduced in 2010, it was suggested that cohort participants, in addition to assessing eligibility based on the presence of symptoms or problems, could be presented with a list of possible interventions, as part of regular cohort data collection. These so-called signaling items assess if they would agree to use an intervention if offered, as we did in the current feasibility trial. The results of the present study and our previous SPIN-HAND trial [39] suggest that, despite selecting patients based on their indicated interest in the cohort’s signaling item, uptake of the offer to try interventions and use among consenters remains low. In intention-to-treat (ITT) analyses, patients who do not accept the offer to try the SPIN-SELF Program are included in the intervention arm. This allows estimation of the effects associated with offering the intervention but does not provide an estimate of the effects among people who are interested in using the intervention, which may be the main effect of interest for some stakeholders [19, 40]. SPIN works with patient organizations to disseminate interventions post-trials, free-of-charge, via their websites, and they are interested in effects among people seeking such opportunities. While estimates of effectiveness among consenters could be improved by statistical approaches such as a complier average causal effect (CACE) analysis [41], this approach would not account for the low usage of the intervention.

We plan to make two major changes to the design of the planned SPIN-SELF Trial, which we will test in an additional feasibility trial with progression to a full-scale trial. First, rather than utilizing the cmRCT design, we will conduct a conventional parallel-groups RCT, utilizing the SPIN Cohort as well as external enrollment procedures to enroll participants. Thus, eligible SPIN Cohort participants, based on their regular assessments, will be contacted and asked to consent to participate in the SPIN-SELF Trial prior to enrollment and randomization. We will additionally increase our recruitment efforts by advertising the trial through social media (Twitter and Facebook) posts to generate interest in the trial from both SPIN Cohort and non-Cohort individuals with SSc.

Second, we will move from an individual self-guided program to a group-based program in which participants use the online SPIN-SELF Program as the curriculum in an interactive program. Low usage of self-guided, online psychological programs, as we encountered in our study, is a well-known problem across settings [42, 43]. While the optimal format and dose of guidance have not been established, there are indications that adding some form of guidance to online interventions improves usage attrition [44, 45]. Various models have been utilized for delivering self-management interventions, including in-person peer-led groups, such as in the Chronic Disease Self-management Program [3] and the Arthritis Self-management Program [46]. An online version of the Chronic Disease Self-management Program, which included email facilitation by peers, had a high level of participation (*N* = 249; 92% logged in at least once; mean logins 25.4, SD 1.7) [47]. Recently, SPIN conducted a trial of the COVID-19 Home-isolation Activities Together (SPIN-CHAT) Program during the first wave of COVID-19 (*N* = 172) [48, 49]. SPIN-CHAT was a 4-week (3 sessions per week) videoconference-based group intervention that provided (a) education and practice with mental health coping strategies and (b) social support to reduce isolation. Of the 86 participants allocated to an intervention group, 75 attended at least one session, and 66 (77%) attended at least 8 sessions. In addition to SPIN-CHAT, SPIN has also used videoconference-based intervention delivery successfully in an almost complete trial of a training program for peer support group leaders [50]. Thus, to address the low uptake of the SPIN-SELF Program, we will add peer-facilitated videoconference-based groups in the full-scale trial, where 8–10 people with SSc will be provided with a directed and interactive experience to support their use of the online SPIN-SELF Program. Intervention groups will be facilitated by trained peer support group leaders to be potentially scalable and because of patient preference for peer support.

The present study has limitations that should be considered in interpreting its results. First, the SPIN Cohort constitutes a convenience sample of SSc patients receiving treatment at a SPIN recruiting center, and patients at these centers may differ from those in other settings. Additionally, SSc patients in the SPIN Cohort complete questionnaires online, which may further limit the generalizability of findings, as all participants already have Internet access and are comfortable using it in a research setting. Third, we were only able to include English-speaking SPIN Cohort participants in this study. French-speaking patients were not included because there was a limited number of French-speaking SPIN Cohort participants at the start of this study, and we wanted to be able to assess feasibility aspects but be able to maximize the number of French participants eligible for the subsequent full-scale trial. At this moment, the SPIN-SELF Program is not yet available in Spanish, meaning we cannot include Spanish-speaking Cohort patients in the trial. Finally, we did not attempt to assess the reasons for not accessing the intervention in non-consenting participants, which might have given us additional insights into reasons for the low uptake.

In sum, the SPIN-SELF feasibility trial identified major problems, including low uptake of the intervention offer among participants randomized to intervention and low usage among those who did consent to try the intervention. This will lead to substantial changes that we will incorporate into a planned additional feasibility trial with progression to a full-scale trial. These changes include a transition from the cmRCT design to a conventional RCT design and a change in the mode of delivery to include additional peer-facilitated videoconference-based groups to the online program. The full-scale RCT will provide information on effectiveness of the SPIN-SELF Program. After the trial, it will be made available through patient organizations around the world to support people in their efforts to cope with living with SSc.

## Data Availability

All data extracted and analyzed for the present study are available from the corresponding author.

## Appendix

### SPIN-SELF Program participant interviews

Process

1. Did the initial invitation email provide you with the information you needed to understand how to sign up for the study?

Yes. No.

If No: What information was missing?

2. Did you find the follow-up telephone call you received within 48 hours of the invitation email to be helpful?

Yes. No.

If No: Why not?

Purpose

3. Did you understand the objective of the SPIN-SELF program?

Yes. No.

If No: How could the objective be clarified?

4. Did you find the information provided in the SPIN-SELF program relevant to you?

Yes. No.

If No: How could the information provided be made more relevant to you or other scleroderma patients?

Words and language

5. Did you find that the program used common, everyday language that was easy to understand?

Yes. No.

If No: Can you give an example of concept(s) or word(s) that you did not understand?

6. Did you understand all the medical terms or, if not, were they clearly explained in the SPIN-SELF program?

Yes. No.

If No: Can you give an example of medical term(s) that you did not understand?

Content, organization, navigation

7. Did you find that the SPIN-SELF program was broken down into manageable chunks or sections?

Yes. No.

If No: Which parts of the content weren’t broken down into manageable chunks or sections and how could we improve them?

8. Did you find the different pages or sections of the program clearly labelled?

Yes. No.

If No: What section(s) could be more clearly labeled?

9. Did you find it easy to navigate through the intervention and to understand where to go next?

Yes. No.

If No: How could the different steps to navigate the intervention be more clearly explained?

10. Did you consult the “More info” tab (Scleroderma 101, Patient Stories)?

Yes. No.

If No: Why not?

11. Did you experience any technical difficulties while using the program?

Yes. No.

If Yes: What type of technical problems? Did you request assistance from the SPIN team? If you did, was the SPIN team able to help you resolve them?

12. Did you use the website tour?

Yes. No.

If Yes: Was it helpful to learn to navigate the website? Why or why not?

13. Did you use the “My Bookmarks” feature?

Yes. No.

If Yes: Did you find it helpful for easily navigating to the pages you wanted? Why or why not?

Learning aids

14. Did the fact that the program was introduced by scleroderma experts and patients make the program more credible and relatable?

Why or why not?

15. Did you understand how to correctly use the techniques explained in the modules?

Yes. No.

If No: What would have helped you better understand how to correctly perform the techniques?

16. Were you able to clearly understand the people speaking in the videos?

Yes. No.

If No: Why couldn’t you understand the words in the videos? (e.g. too fast, too soft, mumbling, accent)? Are there any videos in particular that were more difficult to understand than others? If yes, which one(s)?

17. Did you look at the video transcripts?

Yes. No.

If Yes: Were the video transcripts helpful to you? Why or why not?

Actionability (worksheets, goal-setting, motivation)

18. Did you use the worksheets?

Yes. No.

If No: Why not? What could have been a better tool?

19. Did you set goals for yourself using the “My Goals” feature?

Yes. No.

Why or why not?

20. Did you use the option to share your goals with friends and family via email?

Yes. No.

If Yes: Did the option to share your goals with friends and family via email help you stick with your goals? Yes. No.

If No: What other motivational feature(s) might have been more helpful?

21. Did you incorporate the tools and techniques you learned into your planned routine and stick with it?

Yes. No.

If No: What were some obstacles you faced when trying to incorporate the tools and techniques into your routine? How could the SPIN-SELF program have helped you to overcome these obstacles?

22. Did you use the feature to track your progress?

Yes. No.

If Yes: Did having the option to track your progress week after week encourage you to continue performing the techniques? Yes. No.

If No: Why not? Did you use any other way to track your progress? If so, what did you do?

23. Did you set email reminders for yourself?

Yes. No.

If Yes: Did having the option set email reminders for yourself help you incorporate the techniques into your routine? Yes. No.

If No: Did you use another type of reminder?

Overall evaluation

24. How user-friendly on a 0–10 scale (0, being the worst and 10 being the best possible score) would you rate the SPIN-SELF program?

25. Would you recommend this program to someone with scleroderma?

Yes. No.

If no, why?

26. What grade (on a 0-10 scale, 0 being the worst and 10 being the best possible score) would you give the program?

0 (worst) to 10 (best).

27. Is there anything you want to give us feedback about that was not included in this interview?

## Abbreviations

SPIN: Scleroderma Patient-centered Intervention Network
SSc: Systemic sclerosis
SPIN-SELF: Scleroderma Patient-centered Intervention Network Self-management Program
RCT: Randomized controlled trial
SEMCD: Self-Efficacy for Managing Chronic Disease Scale SEMCD
cmRCT: Cohort multiple RCT
CONSORT: Consolidated Standards of Reporting Trials
ACR: American College of Rheumatology
EULAR: European League Against Rheumatism
PEMAT: Patient Education Materials Assessment Tool for Audiovisual Materials
PROMIS-29: Patient-Reported Outcomes Measurement Information System
ITT: Intention-to-treat
CACE: Complier average causal effect
